# Selections and Abstracts

**Published:** 1901-03

**Authors:** 


### SELECTIONS AND ABSTRACTS.

### SEQUELÆ OF SCARLET FEVER: PREVENTION AND TREATMENT.*

The clinical aspects of scarlet fever are varied; at times it is a complaint so trivial that the patient is not confined to the bed or the house, taxing to the utmost the skill of the diagnostician, the diagnosis being more difficult than the treatment. Again it is seen as one of the most malignant of acute diseases, striking the patient down as if by a blow; in such the diagnosis may be easy, but treatment of no avail. Not infrequently the sequelæ are more disastrous in the consequences and lasting impressions than is the disease itself, often following in the wake of a light attack. We have only to call to mind many of the blind, deaf and dumb, the halt and lame, the unsightly and disfiguring scars about the neck, and instances of chronic Bright's disease attributable too often to the sequelæ of scarlatina; many times after convalescence is well established, and all thought of danger passed, do these sequelæ first present themselves. A difficult problem indeed is to impress upon the minds of the parents and nurses of these little patients the imperative necessity of watchful care and attention long after the bolder symptoms have passed.

While it is true that scarlet fever is not materially modified in its severity nor shortened in its course by any specific treatment, there is very much that may be done by the attendants in preventing the development of its sequelæ. When possible, the physician in attendance should have a specialist associated with him in the management of severe cases. When such desirable association is not at hand, the doctor should be a self-constituted specialist and give his personal attention to such details in the management as will promote a complete and perfect cure. Some of these sequelæ are preventable, some may be modified in their severity, some can not be

*Read before the Louisville Medico-Chirurgical Society, December 7, 1900, by John G. Cecil, B.S., M.D. Professor Materia Medica, Therapeutics, and Public Hygiene, University of Louisville.

controlled nor prevented, but assuredly none should occur through neglect to use every precautionary measure known to medical science.

It is a well-known but none the less lamentable fact that the laity do not appreciate preventive medicine or measures. This is not only true of individuals, but of States and municipalities as well. It is neither necessary nor possible in this paper that all the sequelæ of scarlet fever should be enumerated; it will be more profitable to confine attention to the more common and important.

*Renal Complications and Sequelæ.* The cloudy swelling of the kidneys, with greatly diminished quantity of urine, which is albuminous, of high specific gravty and loaded with urates, phosphates, etc., is constant in early stages of pronounced cases of scarlet fever, as it is in many other acute infectious diseases. This condition should give little uneasiness, as it speedily clears up under the free use of diluent drinks, by the time the febrile symptoms have abated. The acute parenchymatous nephritis which so frequently develops from two to eight weeks after the subsidence of the fever and the disappearance of the rash is due to a mixed infection, or to this associated with exposure to cold and dampness. It is unnecessary to develop this familiar subject; the result would be a dissertation on acute Bright's disease, and the treatment is the treatment of that disease, no matter how caused. The matter of prevention must engage our attention.

First, we should give the strictest injunctions as to exposures for two or three months after the attack; this is particularly necessary during those winter and spring months when scarlet fever is generally prevalent. The clothing of the patient should receive especial attention; woolen underwear is preferable, and the feet should be well protected. Next, I have great confidence in the protective value of the homely and time-honored practice of inunction with fatty substances. It is my practice, and I have never had reason to regret it, to begin inunction with sweet or olive oil, lard, cocoa-butter, mutton tallow, or vaseline as soon as the rash appears, and to continue its use following the bath for weeks after. It relieves itching, prevents the scales from flying, and protects the surface. After desquamation the skin is tender, sensitive, and very susceptible to chill, and nothing will serve so well in its stead as a protective agent. By controlling the excess of fever, by the use of diuretics and diluent drinks, by the use of mild purgatives, preferably the salines, by the use of disinfecting sprays, gargles, and applications to the ulcerated tonsils, pharynx, and nasal cavity, we can modify the course of the disease to a limited extent, and undoubtedly prevent the absorption of noxious agents which are the source of mixed infection, and which, as stated above, play such an important rôle in the development of kidney trouble. The prognosis in post-scarlatinal nephritis is better than when produced by other common causes; the treatment should be active, vigorous, and persistent along the lines recognized as being efficient.

Cervical adenitis and middle-ear disease can, in a measure at least, be prevented and their extension and course modified by zealous care and attention to the throat and nose. The ulcerated throat is almost as characteristic and as constant a manifestation of scarlatina as is the rash. It is from this source that much of the septic absorption takes place, and trouble in the ears, eyes, cervical glands, and the system generally follows. It is well worth the time and trouble to do all that can be done to prevent this absorption. A cleansing spray in the throat and nose of Dobell's solution or any other simple disinfecting solution will do much to prevent the accumulation and absorption of septic material from this source. The application by mop to the ulcerated surfaces of Lœffler's solution or hydrogen peroxide diluted with water and carrying a small per cent. of bichloride of mercury will be of great benefit.

When the cervical glands become tender and swollen, as will often happen, despite our endeavors to prevent, hot fomentations, poultices, the mercurial or iodide of potash ointment will generally cause their dissipation. If suppuration takes place, then the gland must be opened as soon as pus present can be diagnosed.

When the middle ear suppurates, the drum membrane should be punctured under cocain as soon as this fact is determined. The auditory canal must be kept free and open, free drainage encouraged and maintained. The assistance of a specialist is very important and necessary in situations of this kind.

The eyes should be protected from strong light; reading or any close application of sight must be prohibited during height of fever and for some weeks after. Should inflammation set up, soothing lotions and disinfectants must be used with persistent regularity and thoroughness.

Scarlatinal arthritis, or, as it is more commonly called, scarlatinal rheumatism, is not really a rheumatism, but a condition similar to the so-called gonorrheal rheumatism. It is probably an acute arthritis produced by the mixed infection and not by a true rheumatic poison. It is likely to be mon-articular, and shows a decided tendency to suppuration, in these respects differing from the course of true rheumatism. If we can prevent the mixed infection, we probably will forestall the arthritis. In the event of its occurrence the treatment is identical with similar conditions of other origin. The joint should be freely opened and drained.

Heart weakness and failure become at times urgent and alarming. It is my practice to forestall this by the administration of strychnin, iron and quinin, and whisky in nearly all serious cases when any tendency of this nature is manifested. It is well to begin the use of such remedies during the course of the fever or at the time of its subsidence. Not infrequently during desquamation for two or three weeks after the acute stages are past we have a little fever, generally of the septic type, which often continues with annoying persistence, and is not infrequently misdiagnosed as malarial or typhoid fever. Diligent and painstaking search most often shows this to be due to suppuration in the nasal, post-nasal spaces, deep-seated cervical glands, or in the middle ear; appropriate treatment as before indicated will usually suffice to check it.

Of nervous complications chorea occasionally comes on in association with heart and joint troubles. It is probable that this same mixed infection is responsible for this, as it is of so many other sequelæ. Hence allow me once more to urge the importance of prevention by the care that is bestowed upon free elimination of poisons from all emunctories, and by the persistent use of cleansing and disinfecting agents. Chorea is to be treated by prolonged use of arsenic, iron and strychnin.

### PNEUMONIA IN CHILDREN.*

I did not fully realize until I began to think over the subject of pneumonia in children what a task I had undertaken. The subject is so important and interesting to the general practitioner that it should be treated of at length and in a systematic manner; but I am positive that to attempt to bring the subject before you in the manner mentioned in text-books would not only consume too much of your time, but require a more experienced writer than your humble servant.

Pneumonia of the very young is, according to all recent writers, called bronchopneumonia, the term indicating the structures involved. The disease is frequently a primary trouble, but usually a sequel to either measles, whooping-cough, or diphtheria. On account of its frequent association with these other diseases it is very often overlooked, and even when existing primarily it may be overshadowed by prominent cerebral symptoms. The disease is of such frequency, especially during certain seasons of the year, and having for its choice the debilitated children, that it should be a routine practice to examine the chest of every patient, even though the symptoms of pneumonia may not be present.

The causes of the disease, according to writers of recent date, of course vary, although it is almost the universal opinion that the microbe is the mischief-maker.

The symptoms are sometimes, and in fact nearly always, irregular. The attack is frequently preceded for two or three days with a slight bronchitis, slight elevation of temperature, rapid pulse, and hurried respiration. Auscultation at this time will not as a rule show any of the sounds peculiar to pneumonia, but later on we find the little patient with a high fever, painful respiration, very rapid pulse, and crepitant or subcrepitant râles at the base of one or both lungs.

The symptoms as stated above vary, giving us hope of quick recovery when we find the temperature lower, but at the next visit we find the pulse and temperature again higher, with great restlessness and thirst, moaning respiration, etc., and it is then that we con-

* Read by B. A. Allan, M.D., before the West End Medical Society, Louisville, Ky.

clude that we have to deal with a case of bronchopneumonia. We do not find the above symptoms in lobular or croupous pneumonia, but would expect a return to the normal temperature and pulse-rate to indicate the crisis, which we may not look for in bronchopneumonia, but that the disease, if recovery takes place, ends by lysis.

The temperature curve in these cases has according to my observation been varied. I do not regard a high temperature, say 104° or 105° F., as serious; in fact, my experience is in accordance with several writers, that the fatal cases of bronchopneumonia are those that never have a temperature of over 101° F. It never alarms me to have an elevated temperature in pneumonia unless it is too persistent.

The recent epidemic of measles and of whooping-cough in this city has afforded some of us opportunity to observe the diseases followed by or complicated with bronchopneumonia. That in this climate we have in all of these diseases peculiar to children a malarial complication cannot be questioned, if we regard quinine as a test and have noticed a pronounced periodicity in regard to the temperature.

The diagnosis is not always as easy as one would imagine. No doubt many of you have deferred giving an opinion to the family, while suspecting pneumonia and making vigorous search for it at each visit for two or three days without detecting a single physical sign either by percussion or auscultation, until you have been finally rewarded for your trouble by hearing fine crepitant râles, perhaps over both lungs. There is nothing in my opinion which at certain times is more perplexing to the physician than making a diagnosis of pneumonia, especially in children. There is nothing, excepting possibly typhoid fever, which presents such a varied number of symptoms. But when we find it we generally know it, and it is quite a satisfaction to be able to find pneumonia, especially when we cannot make a diagnosis of anything else. An acute onset with persistent high temperature should always lead us to suspect pneumonia, and when in addition we have prostration and perhaps cyanosis the diagnosis is almost certain. Cases of the acute congestive type are the ones most frequently overlooked, and in many instances a positive diagnosis cannot be made; only the physical signs can positively settle the diagnosis. It has been my experience that many cases of infantile pneumonia can exist without consolidation.

The prognosis in bronchopneumonia is usually unfavorable, and it is always best to give a very guarded opinion as to the result. Much depends upon the previous vigor of the patient, and his environment; the tubercular, the rickety, or strumous diathesis, of course, is a factor in causing greater mortality.

As to treatment, every case of pneumonia, so far as its management is concerned, is a law unto itself. Prophylactic treatment is to treat cases of bronchitis with the view of its being a forerunner of pneumonia. It is said by Holt that bronchopneumonia is a very rare disease among the upper class of people.

A patient suffering from pneumonia should first be placed, if possible, in an airy, well ventilated, and well lighted room. If a stove is used for heat, it is important to keep a kettle of water constantly steaming, as a dry atmosphere with poor ventilation is said to deprive the patient of much needed oxygen. The clothing should be a flannel gown; the patient should be kept in bed rather than on the lap of the nurse; a cotton-batting flannel and oiled silk jacket should envelop the entire chest after having previously used inunction of camphorated oil and turpentine; the hips of the patient should be slightly elevated, favoring the blood-supply to the lungs and heart, and thereby favoring the expectoration, etc.; cold water in abundance and *ad libitum* is essential. Cold water is not only grateful to the patient, but it is my opinion that it is what might be termed nature's expectorant as well as beverage. I usually insist upon my patients taking large quantities of water, either in the form of orangeade, lemonade, or pineappleade, with white of egg raw; stimulants in the form of whisky, brandy, or sherry wine, especially if the pneumonia is secondary, are quite necessary.

As to therapeutics, I am inclined to treat my patients symptomatically, or on the expectant plan. Just because a case is diagnosticated as pneumonia, and in the congestive stage, it is my opinion that the routine of giving carbonate of ammonium, digitalis, etc., should not be resorted to. It is best, in my judgment, to give nothing save minute doses of calomel in the beginning, followed by small doses of aconite, combined with acetate of potassium and spirits of nitre, when other remedies such as muriate of ammonium or possibly carbonate of ammonium in the form of spirit of mindererus might be indicated. But I candidly believe many cases would recover without medicine, with good nursing and proper hygienic surroundings, a mustard foot-bath, cold drinks, a dose of bromide to relieve nervousness, a sponge bath of alcohol or vinegar and water under cover if temperature is persistently high.

The traditional poultice in my judgment has yet an occasional place in the treatment of pneumonia in children. On several occasions have I seen cases with continuous high temperature, nervousness, sleeplessness, crying, and moaning, and with pleasure witnessed a magical improvement within an hour after the application of a flaxseed poultice with which was incorporated a teaspoonful of mustard, and this covered with oiled silk.

The progressive trend of modern therapeutics should lead us to at least hope that serum therapy will find a wide field of usefulness in the treatment of pneumonia in children, and I trust that these little sufferers may in the near future be spared by the use of an antitoxin as efficacious as diphtheria antitoxin.—*Medical Age.*

---

### THE TREATMENT OF INFLUENZA.*

By WM. H. THOMSON, M.D., LL.D.

The author, who has had a severe attack of la grippe, and who has treated numerous cases in personal practice and in consultation, has elaborated a plan of treatment which has proved highly successful. For the general aching pains that characterize the onset of this disease, as of so many other febrile affections, aconite is the best remedy. Not only is it one of the most effective analgesics for this class of pains, but it counteracts the accompanying vascular derangements, particularly of the upper respiratory tract. The author believes that he often aborted an acute tonsillitis by the early administration of a dose of tincture of aconite, sufficient to cause the characteristic sense of constriction in the throat. At the beginning of febrile infections, including that of croupous pneumonia, aconite seems not only to relieve the systemic pains,

* Read before the N. Y. Acad. of Med., Jan. 15.

but to modify for the better any local capillary stasis setting in with the onset of the fever. The heart, under its influence, becomes much quieter and the pulse softer. This beneficial action of aconite seems to be further promoted by the addition of a small dose of Dover's powder. Another drug which the author has found very serviceable is phenacetin, and its analgesic action, especially when combined with aconite, is more marked in influenza than in any other disease. It should not be given in doses sufficient to reduce temperature, because temperature in influenza is a very secondary matter; and, moreover, in antipyretic doses it may have the dangerous effect of weakening muscular action. But when given in small doses, combined with quinine, it may, in the author's belief, act as a true antitoxin to the influenza poison. The author's experience is decidedly in favor of this hypothesis, for these two drugs have seemed to mitigate the symptoms and to prevent the sequelæ of the disease, evidently not by their antipyretic properties, but by a specific neutralization of its effects, as indicated by the less favorable course of those cases in which phenacetin and quinine have not been administered.

Coming to detailed treatment, the author prescribes pills each containing the following ingredients:

| | |
|---|---|
| Extract aconite | 1-12 grn |
| Dover's powder | ½ grn |
| Phenacetin | 2 grn |
| Quinine sulphate | 1½ grn |

For one pill. Two such pills to be taken three times a day, as long as any febrile temperature remains. As soon as the temperature declines, whether it be on the second or a subsequent day, the dose is reduced by one pill a day, till only three are taken and this is continued until all catarrhal symptoms have subsided. In patients too susceptible to the action of aconite, the dose of the latter may be reduced.

In certain types of the disease, coryza, nasal catarrh and tracheitis are leading symptoms and in these conditions a pill of a quarter of a grain of extract of belladonna with a grain or two of camphor acts beneficially; an additional useful measure is flushing of the throat by the aid of a fountain or bulb syringe with a quart of hot water, in which two teaspoonfuls of potassium chlorate and five drops of oil of peppermint have been dissolved. This often affords marked relief by causing a great flow of mucus from both nose and throat. Where the supraorbital sinuses become involved, accompanied by excruciating pain and often photophobia, ergot is indicated. It is specific in its action and the fluid extract should be given in dram doses, repeated every three hours if necessary; adding a teaspoonful of elixir of cinchona to each dose makes it more acceptable to the stomach.

A markedly paroxysmal dry cough, which has nothing to do with bronchitis, but is evidently of a nervous character, sometimes makes its appearance during an attack of influenza; it may persist for weeks after other symptoms have subsided. It is apt to be especially troublesome at night. This cough generally yields to ammonium bromide and antipyrine: 20 grains of the former to 10 of the latter.

We now come to the treatment of the respiratory features of influenza. A bronchitis in the course of influenza sometimes becomes a very serious affair, especially in patients past middle life, with more or less endarteritis and a high-tension pulse. It generally makes its appearance after a week or more of the ordinary catarrhal symptoms, the temperature rising rather suddenly from 99° to 102° or 103° F. The bronchitis is quite obstinate; ordinary remedies bring but temporary relief. After a time signs of respiratory failure may develop, with the constant presence of râles at both bases; the power of expectoration is diminished and the bronchitis is really converted into a bronchopneumonia. This bronchopneumonia may be said to be caused by the occlusion of the smaller bronchi with plugs of tough viscid mucus and not by an extension of the catarrhal process itself from the bronchi to the air lobules. The indications, therefore, are to liquefy the retained secretions as speedily as possible, stimulating at the same time the function of expectoration. The ammonia compounds, useful as they are in inflammation of the trachea and larger bronchi, are singularly ineffective as expectorants when the inflammation has reached the smaller tubes. For this condition, as well as converting a viscid secretion into a thin fluid, the best remedy is the author's linseed emulsion, which he has been advocating for the past twenty-five years.

The author wishes, emphatically, to emphasize the point that he knows of no so-called expectorant in bronchitis which equals this combination; he adds to each dose of emulsion $\frac{1}{12}$ grn. of morphine and 8 grn. of chloral to allay the nervous elements of irregular bronchial muscular action. Counterirritation is a very important adjunct to the treatment of bronchitis. This consists, first, in a thorough cupping of the chest, both anteriorly and posteriorly; the weak cupping by glasses with rubber bulbs is wholly useless. Following the cupping the author advises application to the chest of large cloths, wet with an infusion of capsicum made with a dram of the drug to a pint of boiling water; this is preferable to mustard, as it causes no vesication or sores. It is well to keep up the counterirritation for a long time, and a good liniment for the purpose is the following:

| | |
|---|---|
| Ammonia water | of each, 1 oz. |
| Oil turpentine | of each, 1 oz. |
| Tincture capsicum | of each, 1 oz. |
| Soap liniment | 3 oz. |

For the debility which follows influenza the author relies mainly on the fluid extract of coca and nux vomica. Inhalation of oxygen is also often useful if administered with a funnel over the nose and mouth as in administering ether.

Besides all the above measures, rest in bed, until permitted to get up by the physician, is of paramount importance.

---

BIRTH-RATES, ALCOHOLISM, AND RURAL AND URBAN LIFE.

Some marked and highly important changes have been noted by statisticians, bearing on national life in several fields, which do not appear to the casual observer to have any particular relations to one another. The decline in birth-rates when compared with death-rates in several European nations, the great increase in alcoholism everywhere, and the remarkable flow of inhabitants from country to city, constitute a problem in the lives of races and nations of the most profound portension.

The narrow margin between the death-rate and the birth-rate existing in France has been familiar for some time. Recent statistics of England begin to show a tendency, to say the least, in the same direction. The last report for London shows the lowest birth-rate yet given—29.1 per thousand. At the same time the death-rate (21.1 per thousand) is lower than usual. The death-rate of infants under one year of age varied in different parts of the city from 112 to 225 per 1,000. There has been a steady decrease in the margin of per cent. between births and deaths (at present 7.2 per cent.). Similar changes are going on in the nation as a whole.

The cause of birth-rate decline in France has usually been attributed to various traits more or less peculiar to its people, the indifference to offspring, to the marriage law, to the use of highly stimulating forms of alcohol, absinthe, vermouth, and liquors, and comparative indifference to athletics; but when similar birth-rates begin to suggest themselves in sturdy old England, the home of rosy-cheeked, rotund lovers of roast beef and port, we begin to reconsider the question of human reproduction in its economic sense.

A recent paper by Dr. Wm. Carter gives the general death-rate of Great Britain from 1837 to 1872 as being without change, but from 22.5 per 1,000 in 1872 it has fallen to 17.2. Classification study of these figures show that of the ten general divisions of diseases and causes of death there has been a steady decline in the death-rate in nine of the ten, and the exception is in the division of intemperance. Here there has been an increase from 45 deaths per 1,000 in 1872 to 77 in 1897; but for this the death-rate would have fallen to 14 per 1,000.

In the November issue of *Obstetrics* we publish an editorial on "Wine-Drinking during Pregnancy," in which, we think, we showed the very direct intoxication of the fetus which must result from use of liquors by pregnant women. The weakening of vital forces, forces which are at the foundation of reproductive power, by the rapidly extending use of alcohol among the great working classes is beginning to show its effect. Absolute drunkenness was perhaps more common formerly than now, but the actual consumption per capita is now much greater.

The second great national influence which, we believe, is affecting the birth-rate is the remarkable movement of the people from the rural and small-town districts to the large cities. We know what that movement is in the United States and it seems that the same centralizing change is going on in European nations. Recent statistics of Germany's census show a tremendous drift, not tide, for there is no return, from rural and urban residence. The effect of life in a great city upon the well-to-do is not injurious, but upon the class which only works, which gets no country outing as the years roll by, that live in sunless, ill-ventilated, and crowded quarters, ever breathing a vitiated air, is disastrous. We have seen it stated by competent authority that the average life of a family living constantly in large cities without intermarriage with fresh country blood is something less than five generations.

Alcoholism and urbanification, to coin a word, are the two main factors affecting declining birth-rates.

What is the cure? We are somewhat pessimistic about the possibilities of reduction of alcoholism, but for the movement from country to city, we can do much to change the situation from a drift to a tide. There has been, in a very moderate degree, a sending of children of the poor to farms from institutions in large cities; but this is not enough to make much impression against the great influx. Such outward movement should be extended all along the line, Every street arab and child who is self-independent should be encouraged to seek the country. The present efforts at tenement house reform, which aim not only to improve sanitary conditions of such dwellings, but to lessen actual congestion of populations by favoring the removal of manufactories, sweat-shops, and other lines of occupation which can just as well do so, to the suburbs, are of the utmost value in every way.

It is remarkable how small a per cent. of the city-raised poor ever reach a standing in life of notable credit; whereas, it is equally remarkable how large the number of the leaders in the higher walks of city life were brought up in comparatively rural surroundings.

Alcoholism prevails in the country as well as in the city, but is more confined to occasional sprees by a few, with much lower per capita use of alcoholics than prevails in the cities. The total per capita consumption of alcoholics is approximately one half that of England on the part of Russians with a rural population of 90 per cent. of the whole living outside cities as compared with 61 per cent. in England.

Again we say we must make the drift a tide.—*Obstetrics.*

---

### TREATMENT OF PROSTATIC HYPERTROPHY.

Guiteras (*New York Medical Journal,* vol. lxxii, No. 23) is in the habit of performing prostatectomy by what he calls the recto-vesical method, although no part of the operation proper is performed through the rectum, the fingers simply being inserted to exert direct counter-pressure and guide the operating finger, which is working through the bladder. The steps of the operation are, briefly, as follows: Suprapubic cystotomy is performed, after which retention sutures are passed through the bladder wall on each side of the incision. Careful rectovesical palpation is then made, two fingers of the left hand being in the rectum, while the right fore-finger is in the bladder, and a visual examination is made by means of an electric light inserted into the bladder. A pair of scissors is inserted into the bladder, the closed blades being pressed against its floor in the region of the neck in the median line, until the blades are felt depressing the prostate against the two fingers of the left hand in the rectum, which are in turn pressing upon the gland in the median line from below, showing exactly whether they are in the right position or not. The blades of the scissors are opened, and a cut is made through the bladder tissue covering the gland in a line corresponding to the space between the fingers and the rectum. The forefinger of the right hand is then inserted in this cut through the bladder, and gradually works its way between the capsule and the gland. The two fingers in the rectum feel the one in the bladder working its way between the capsule and the gland, and they start counter-pressure while the enucleation is being performed. The finger-tip under the capsule first sweeps to one side, and pulls out one lateral lobe, then to the other side, and enucleates the other, and finally removes the middle lobe; or it first works its way backward under the base of the middle lobe, which is enucleated with a dissecting rotary movement, and then proceeds to enucleate the two lateral lobes. The gland having been removed, there is always free hemorrhage, and very hot water should be injected for two or three minutes into the bladder suprapubically, during which time the surgeon again washes his hands. He then passes a grooved sound, preferably a lithotomy guide, through the urethra into the bladder, and having brought the patient into the lithotomy posture, he rapidly cuts through the membraneous urethra, and pushes a large peritoneal drainage-tube into the bladder, which he fastens to one side of the perineal incision. The finishing steps of the operation are to put two catheters into the bladder suprapubically, with a gauze drain packed down beside them into the cavity made by the removal of the prostate, fasten them into the skin of the bladder wall, and then sew the bladder wall up the drain. The surgeon then closes the abdominal wall, including fascia, muscle and skin, as far as the drain. The floor of the prostatic urethra should be cut through from above downward before the perineal tube is left permanently in place, to prevent the formation of a pocket, which would retard the cure.

*The After-treatment.*—An enema of a pint of hot saline solution at a temperature of 120° F., to be retained, should then be given to the patient, together with one-thirtieth of a grain of strychnine, and he should be put to bed with hot bottles at his feet. The shock in these cases is often great, and should be guarded against as much as possible. As soon as he comes out of the effects of the ether, a little hot water or bouillon should be given, and from this time on the hot water should be pushed *ad libitum.* If the patient vomits the water, give more in a few minutes, and continue to force it. It is wonderful how much water can be given in these cases, some patients taking between one and two gallons in the first twelve hours. This has the effect of flushing the kidneys and producing free diuresis. Any water is good for the purpose, although Dr. Guiteras is in the habit of prescribing some mild diuretic spring water.

The strychnine in one-thirieth-grain doses should be given every four hours hypodermically, and it is well to repeat the hot saline enema every four hours. In this way something can be done for the patient every hour if they are alternated, either strychnine, hot rectals, bouillon, or water, remembering that the water should be pushed to its maximum tolerance.

It is well, both before and after operation, to give a urinary antiseptic, preferably urotropin, in ten-grain doses, three times a day. A diet of milk, from two to three quarts a day, is advisable. The bowels should be moved with some saline water, Apenta or Hunyadi, on the second day.

The suprapubic tubes can be removed at the end of a week, and the perineal one at the end of two or three weeks. The drainage in these cases is by the siphon method, into bottles hanging from the side of the bed, two pieces of tubing going from the suprapubic opening, and one from the perineal.

In closing his remarks, Dr. Guiteras states that he considers the operation of prostatectomy as still in its infancy, in the same position as hysterectomy was some years ago, but he has no doubt that it will some day be simplified, and it behooves all surgeons interested in this line of work to try to improve the methods and technique of the procedure.—*Therapeutic Gazette.*

---

### FOLLICULAR TONSILLITIS.

BY GEO. A. HEWITT, M.D., PHILADELPHIA, PA.

In this disease, so common among young children, Glyco-Thymoline (Kress) may be employed with advantage. Its exosmotic properties are extremely serviceable in reducing the size of the swollen glands. The enlargement is apt to subside slowly, even after the active stage of the inflammation has passed. Children who are old enough to gargle may employ the remedy in this manner. In those too young it may be applied upon absorbent cotton. Representative cases are :

Case 1.—A boy, eleven years of age, had suffered for a day from headache and fever, with pain in swallowing. Both tonsils were red and swollen, especially the right, which was studded with patches of exudation from the crypts.

Case 2.—A girl, age fifteen years, had had a chill twenty-four hours previously, her throat felt sore, and it pained her to swallow. The glands of the neck were swollen and there was fever. Both tonsils were considerably enlarged. The crypts were exuding their characteristic discharge.

Case 3.—A young woman, nineteen years of age, was attacked by vertigo and lost consciousness. Twelve hours later both tonsils were found greatly swollen, almost meeting in the middle line. The cervical glands were moderately enlarged. The surface of the tonsils was dotted by exudation.

These three cases were all treated successfully with Glyco-Thymoline (Kress) as described above.

CHICAGO, ILL.

P. C. Madison, M.D., writes:

I take great pleasure in reporting to you the splendid results obtained in using your preparation, Glyco-Thymoline (Kress) in the treatment of diseases of the nose and throat. Used in compressed air applications, and also with your Bermingham Nasal Douche.

Case 1.—Patient Ruby B . . . , age ten years, had been treated for two years by the family physician for chronic bronchitis, hypertrophied tonsils and loss of voice. The mother of the child objected seriously to have the tonsils removed, and prevailed upon me to try medical treatment instead. Result as follows:

Used Glyco-Thymoline (Kress) reduced to one-third, spray for throat, same strength for nasal douche. Did not use internal medication, except first month, quinine, iron and strychnine. Last three months no internal medication. In forty-five days from first treatment tonsils were reduced to normal size. Nasal and pharyngeal catarrh almost entirely relieved, and voice much improved; treatment then continued, sprays and douche only, to end of fourth month, at which time I dismissed the case, the nose and throat trouble having entirely disappeared, also perfect restoration of voice.

"TONSILLITIS."

PHILADELPHIA, PA.

J. S. Miller, M.D., reports following cases:

Case 1.—Girl, fourteen years. Follicular tonsillitis; she was very anemic, large quantities of cheesy matter; both tonsils badly affected, sprayed the throat with I. O. and afterwards followed by spray 50 per cent. solution of Glyco-Thymoline (Kress); the recovery was very rapid.

Case 2.—Mrs. R . . . , aged twenty-two years. Peritonsillar abscess. Opened it and washed out the cavity with Glyco-Thymoline (Kress) and ordered gargle of the same 50 per cent. solution; result was very satisfactory.

CINCINNATI, O.

M. E. B. Thompson, M.D., states: I was taken ill with a case of follicular tonsillitis one week ago, gargled with Glyco-Thymoline (Kress) every three hours; seemed to act as a specific. Fever subsided, allayed inflammation and it yielded to Glyco-Thymoline (Kress).

CLEVELAND, O.

E. U. Garvin, M.D., states: I have tried Glyco-Thymoline (Kress) on wound of tonsil produced by a child falling with a tin horn in the mouth. I kept this part thoroughly cleansed with Glyco-Thymoline (Kress); full strength and result was gratifying. I wish to give it a thorough test in catarrhal troubles.

HAMILTON, O.

C. W. Mayer, M.D., reports following cases:

Master K . . . , fifteen years old, came to my office and had a fully developed case of follicular tonsillitis, both sides being involved by the inflammation. Placed him on constitutional treatment for the fever and Glyco-Thymoline (Kress) as a local treatment, by swabbing the affected part with one half strength every one or two hours. The next day to my great surprise all the inflammation and white patches had disappeared, the spray was ordered continued three times a day; in three days the case was all well and sound.

Have treated three cases since that time with uniformly good results.

ALTON, MO.

J. K. Cantrell, M.D., states: I suffered for twenty years from nasal catarrh and at times experienced the most agonizing neuralgic pain of a superior orbital character; like all other physicians thought but little of using any remedy than my own until I received the sample bottle of Glyco-Thymoline (Kress) sent me by you; it set in my office for months, until one of those neuralgic attacks came on, and after using my own remedy with little or no satisfactory results, I commenced the use of Glyco-Thymoline (Kress) and was relieved in eight hours of the neuralgic pain, and I am glad to say I am as free from nasal catarrh as an infant. The above is the only disease I have had a chance to use Glyco-Thymoline (Kress) in, as I have used the sample sent in curing myself. In conclusion will heartily recommend Glyco-Thymoline (Kress) to all who have nasal catarrh. It will cure any case unless the disease is of a syphilitic origin. I think Glyco-Thymoline (Kress) should be introduced in every physician's practice in the United States. I for one shall recommend and use it in every case that comes under my treatment for nasal catarrh.

Kress & Owen Company,
Manufacturers of Manufacturing Chemists,
Glyco-Thymoline (Kress), 221 Fulton street, New York.
K. & O. Bermingham Nasal Douches.

---

### THE TREATMENT OF SYPHILIS. A NEW AND TOLERABLE FORM OF ADMINISTERING MERCURY, WITH REPORT OF SIXTY-FIVE CASES TREATED AT BELLEVUE HOSPITAL.*

BY WINFIELD AYRES, M.D., NEW YORK CITY.

Instructor in Genito-urinary Surgery at the New York Post-Graduate Hospital, and attending Genito-urinary Surgeon at Bellevue Hospital, Out-door Patients' Department.

The writer states that when his attention was called to Mercurol as an antiseptic of special value in the treatment of gonorrhea, it

* Abstracted from the Author's Original Paper in the *Philadelphia Medical Journal*, November 10, 1900.

occurred to him that it would be a first-class preparation for the treatment of syphilis. Some time was necessarily spent in determining the proper dosage. At first one-eighth of a grain was given three times daily, and this dose was gradually increased until it was found that three grains was the average quantity required to control the malady. The highest amount given was seven grains and the lowest amount that exerted a controlling influence upon the disease was one-half grain. In starting a patient on a course of Mercurol the author advises beginning with half grain or grain doses. Salivation has been produced by two grains, and yet as much as six grains has been taken with no disagreeable symptoms.

The objections to the use of unguentum hydrargyri as a remedy in secondary syphilis are referred to; and while the popularity of mercuric protiodide is conceded, the irregularity of its action and its tendency to cause gastric and intestinal disturbances are not overlooked. In the writer's experience 33 per cent. of his cases were not benefited by this drug.

Mercurol is a nucleid of mercury, and was discovered by Karl Schwickerath of Bonn, Germany. Kopp, Director of the Royal Polyclinic for Genito-urinary Diseases at the University of Munich, uses Mercurol in smaller doses, which leads the writer to remark "he will find, as I have done, that it is desirable to use a much larger dosage." Mercurol should not be given in solution with potassium iodide.

In all, 65 cases received Mercurol at the Bellevue clinic, 60 of which had not had previous treatment. Of these, 13 did not return after the first or second visit; 14 did not remain long enough under treatment to give the preparation a fair trial; and 13 may be described as new patients. Deducting these 40 cases, there remain 25 cases that have been sufficiently long and regular in their attendance to supply data from which definite conclusions may be deducted. The detailed histories of these 25 cases are included in the paper. In summarizing the author remarks that while two months' treatment of syphilis is insufficient to determine absolutely the value of any remedy, the marked improvement shown by many of his cases makes it certain that Mercurol is of great value. Its superiority to mercuric chloride in controlling the symptoms of syphilis is proved. Like all internal remedies it has very little effect upon the initial lesion; still it has hastened the healing slightly. None of the cases required treatment with potassium iodide to control secondary manifestations.

To recapitulate, (1) Mercurol causes less disturbance of the gastro-intestinal tract than any other preparation of mercury used internally. (2) It controls skin eruptions and pains much better than any other preparation, while it controls mucous eruptions as well as any other, and has equally as good an effect upon the chancre. (3) It is an advantage that it can be taken in pill form.

---

### THE USE OF BROMIDES IN HYSTERIA, DELIRIUM, ETC.

BY J. S. MURPHY, M.D., SULLIVAN, IND.

Considerable has been written on this subject which has all the respectability of ancient lineage. And like most other obscure things has received no stint of authoritative attention.

The etiology of hysteria has never been satisfactorily explained. For a long time it was thought to be in some way related to uterine disturbances. But while it is not denied that sexual disorders may have a bearing on the primal cause of the phenomena, still it is also claimed that the ailment attacks both sexes. We have progressed not further than this.

The treatment at best has been attended in most cases with disappointing results. We are confronted with a "loss of due balance between certain of the high functions of the brain, spinal cord and sympathetic system." The treatment obviously should be, then, to restore this balance. Rest is a very essential feature. By rest is meant *restraint* of overaction of certain of the spinal nerve centers. My experience has taught me that nothing gives better results than the combined bromides; and these should be of the very purest obtainable. For this reason I have availed my professional self of Peacock's, not only for their purity—freedom from bromates and carbonates so common to the commercial bromides—but on account of their ideal synergic effects and the fact that they are neutral in reaction, which permits of combining certain alkaloids in the solution without fear or danger of precipitation.

In various forms of neurosis I have found Peacock's Bromides invaluable as an all-round agency of alleviation and cure. They have never disappointed me. In obstinate cases of epilepsy, where the treatment is necessarily protracted, I find them particularly useful in that their administration is not followed by the too common symptoms of bromism. And I would specially urge their utility in instances of delirium following alcoholic excesses.

Anything that conserves the vital forces, that does not depress any organ, as for example, the cardiac center, anything that gives the rest of normal sleep when repair is greater than waste, anything that tends to restore the nervous equilibrium, soothing the exciting centers, whatever and wherever they may be, must benefit the entire organism when each separate organ, then, of course, will receive its needful quota of help. And since local treatment is out of the qustion, I cannot conceive of better procedure, or one more infallible to the successful management of hysterical cases.

---

### SOME SUGGESTIONS ON THE MANNER OF USING PROTARGOL.

Having passed the experimental stage it may now be safely asserted on the ground of the remarkably extensive literature published, that protargol is one of the most important additions to the materia medica of recent years. Aside from its general use in the treatment of gonorrheal affections it has to a great extent displaced nitrate of silver in disease of the eye, ear, nose and throat. To obtain uniformly good results, attention has been lately drawn to the importance of exercising proper care in making the solutions, a point which has been especially emphasized by Professor Neisser. A clear and satisfactory solution can be secured in any one of the following ways: Stir the protargol powder into a thick and smooth paste with a little cold water and then add the bulk of the fluid. This should be done in a glass or china vessel, using glass rod; if in a mortar, the latter as well as the pestle, should be slightly moistened with a few drops of glycerine. Protargol may also be readily dissolved by dusting the powder evenly upon the surface of the water and allowing the fluid to stand without stirring for about ten minutes. It is very essential that only *cold* water should be used in making the solutions, as with warm water the drug is to some extent decomposed, and then becomes less active and may cause irritation; for the same reason the solutions should be preserved in dark colored yellow bottles. In acute gonorrhea the average strength of the solutions ranges from one to ten grains to the ounce; in chronic urethritis, up to thirty grains; in diseases of the eyes, ears, nose and throat, ten to sixty grains; as an application to wounds and ulcers, one to two per cent. solutions and five per cent. ointments are in use. Unlike nitrate of silver protargol does not stain the skin even in concentrated solution. The solutions commonly employed in gonorrhea also do not produce stains of the clothing, or if they do, only cause slight discoloration, which can be easily removed with warm soap water. The much stronger solutions of twenty to fifty per cent. sometimes leave behind brownish-yellow stains on the clothing; if recent, they can be removed with soda and ammonia; if old, by the action of peroxide of hydrogen in the presence of ammonia.

